# Compositional Changes during Storage of Industrially Produced Olive Oils Co-Milled with Olive Leaves

**DOI:** 10.3390/foods13010073

**Published:** 2023-12-24

**Authors:** Anja Novoselić, Tullia Gallina Tosci, Dora Klisović, Matilde Tura, Karolina Brkić Bubola

**Affiliations:** 1 Institute of Agriculture and Tourism, Karla Huguesa 8, 52440 Poreč, Croatia; novoselic.anja@gmail.com (A.N.); karolina@iptpo.hr (K.B.B.); 2 Department of Agricultural and Food Sciences, Alma Mater Studiorum—Università di Bologna, 40126 Bologna, Italy; matilde.tura2@unibo.it; 3 Independent Researcher, 52100 Pula, Croatia; klisovic.d@gmail.com

**Keywords:** olive oil, olive leaves, co-milling, phenolic compounds, volatile compounds, sensory characteristics, oxidative stability

## Abstract

The possibility of enriching the oil produced from Leccino cultivar olive fruits with phenolic and volatile compounds by adding olive leaves (2.5%) during industrial-scale production were investigated. Furthermore, the influence of the addition of leaves on the oil quality, composition, and oxidative stability during storage for 6 and 12 months was studied. A slight negative impact on the oil quality after processing with leaves was determined. The addition of leaves had no influence on the total saturated, monounsaturated, and polyunsaturated fatty acids in oils, while it influenced increases in total phenolic compounds (+29.55%), total secoiridoids (+29.43%), chlorophylls (+47.59%), and the oil stability index (+18.70%), and their higher values were also determined in the stored oils. The addition of leaves increased C5 volatiles (+10.50%) but decreased C6 volatiles (−10.48%). The intensity of most of the positive sensory characteristics increased in fresh oils obtained with leaves, and the established improvements were also maintained in the stored oils. The extractability of olive paste was positively affected by the addition of olive leaves, which increased the oil yield (+27.17%). The obtained results significantly contribute to the knowledge about the possibilities of enriching olive oils with bioactive compounds.

## 1. Introduction

The cultivation of olives and the production of olive oil generates considerable amounts of waste materials and by-products, such as olive pomace or leaves, and the failure to use these residues and by-products results in the loss of potentially useful compounds [1]. Olive leaves accumulate during olive pruning (about 25 kg of branches and leaves per tree in a year) [2], but also in the olive oil processing industry after they are separated from the fruits before processing (3–10% of the weight of the olives depending on the way the olives are harvested) [3,4]. Regarding standard production practices, olive leaves are most often discarded as waste and burned, while only partially used for composting or as animal feed.

Different present studies show the high added value of olive leaves because they are an excellent source of compounds with biological properties [5,6,7,8]. The olive leaf is an excellent source of phenolic compounds [9] and contains relatively high amounts of oleuropein (1–14%) when compared with olive oil (about 0.005–0.12%) [10,11]. In addition to phenolic substances, the leaf also contains different biologically active ingredients, such as chlorophylls, tocopherols, β-carotene, squalene, triterpenes, and sterols [12]. Considering the mentioned composition, olive leaves can be considered a rich natural source of antioxidants due to the high content of phenolic compounds [3], which have a strong preventive antioxidant effect [13,14,15]. Olive leaves represent a cheap and environmentally acceptable source of bioactive compounds [11] and, therefore, experience increasing interest from pharmaceutical and food companies [4,16,17]. Olive leaf extract has strong antioxidant activity, and it is approved by the European Food Safety Agency [18] as a safe product, which opens up the possibility of its use in the food industry. The use of olive leaves as a raw material in the production of antioxidants could contribute to the efficiency of biological waste disposal, which until now mostly relied on ecologically unacceptable incineration [19].

It is known that virgin olive oil (VOO) can degrade in quality under certain conditions of production and storage, especially due to the oxidation of oil [20,21]. Therefore, the production of olive oils with an increased content of phenolic compounds, which can additionally protect the oil from oxidation, is of great importance for the quality and oxidative stability of the product [22]. Until now, many attempts have been made to increase the content of phenolic compounds in olive oil, especially by optimizing the oil extraction conditions, such as the temperature and time of the malaxation of the olive paste [23,24], and, in more recently time, by the addition of natural sources of phenolic compounds, such as by-products from the olive and olive oil production, especially by olive leaf addition [25,26,27]. Although during the production of olive oils a small part of the leaves remains after the cleaning process, it should be emphasized that the intentional addition of a larger quantity of leaves would not be in accordance with EU regulations for virgin olive oils [28] since virgin olive oils are oils produced exclusively from olive fruits without any other additives.

Several studies have been published with the aim of improving the physico-chemical and sensory characteristics of the oil using the addition of olive leaves during the oil extraction in the laboratory or small production scale [29,30,31,32]. Most of the previous studies have been focused on the influence of the addition of leaves on the phenolic compounds and antioxidant activity of the oils but with contradictory results. Some studies reported an increase in the concentration of total phenols and antioxidant activity [25,27,29,30,33], while some reported a decrease or no changes in the content of phenolic compounds [32,34,35] after the addition of leaves. The results obtained in the research conducted on a laboratory scale are not always the same as those conducted on an industrial scale, even though the same malaxation times and temperatures of olive paste are used [36]. The difference between the laboratory and industrial scales is mostly in the batch size and the fact that the olive paste in the laboratory scale is more exposed to oxygen than in the industrial scale, which can lead to differences in the composition of the produced oils due to the different influence on the enzymatic processes involved in the formation of certain phenolic and volatile compounds responsible for the olive oil taste and odor [35]. Therefore, it is important to transfer the research to an industrial scale to be able to clearly show the real conditions of production practices and the characteristics of oils produced on an industrial scale. According to our knowledge, only two studies have been published that applied olive leaf addition during oil production under industrial conditions [26,37], and none of those studies involve the investigation of changes in the oil during storage. Previous research that took into account the phenolic composition of the oil determined that the influence of the leaf addition also depends on the olive leaf cultivar and the proportion of added leaves [25,26,38].

Therefore, this study aimed to investigate the possibility of enriching the oil produced from Leccino cultivar olive fruits with phenolic and volatile compounds using the addition of olive leaves during industrial-scale production. Furthermore, the influence of the addition of olive leaves (2.5%) on the quantity of produced oil, as well as the quality, fatty acid composition, phenolic and volatile compounds, sensory characteristics, and oxidation stability of the oil during the storage, was studied. Due to the lack of data in the literature, this study could significantly contribute to the knowledge about compositional changes during storage and the oxidative stability of oils produced from olive fruits with the addition of olive leaves on an industrial production scale.

## 2. Materials and Methods

### 2.1. Materials

#### 2.1.1. Harvesting of Olive Fruits and Leaves

The study was conducted on leaves and fruits of the Leccino olive cultivar, the most abundant introduced olive cultivar in Croatia, represented also in the assortment of many Mediterranean countries. Leccino cultivar fruit and leaf samples were obtained from a reliable local producer in an olive grove near the city of Poreč (45°26′52″ N; 13°62′97″ E), Istria, Croatia. The olives were harvested manually on October 14, 2019, when the fruits had a ripening index of 3.16 (purple epidermis). The ripening index was determined according to the method of [39], which is based on the evaluation of the colour of the skin and flesh of the fruit. The olive leaves of the Leccino cultivar were harvested by hand on the same day, at the same time, and from the same trees as the olive fruits.

#### 2.1.2. Processing of Olive Fruits

The processing of olives was carried out within 24 h of the olive fruit harvesting. Before processing, olive fruits were cleaned using a washer and defoliator (Oliomio, Toscana Enologica Morri, Italy) to remove existing impurities, branches, and leaves. Milling of fruits with or without added fresh leaves was carried out in a metal crusher with knives (Oliomio, Toscana Enologica Morri, Italy). In order to test the effect of leaf addition on the composition of the obtained oil, the addition of leaves (2.5% of the olive leaves on the weight of the olive fruits, *w*/*w*) was manual and was carried out just before the start of milling the fruits. The rate of olive leaves (2.5%, *w*/*w*) was chosen in order to simulate percentage of olive leaves in olive fruits after hand harvesting and before cleaning of fruits during olive oil production. Malaxation of the olive paste was carried out in a vertically placed stainless steel malaxator (Oliomio, Toscana Enologica Morri, Italy), with a capacity of 300 kg, at 25 ± 1 °C for 30 min. The oil was then separated by centrifugation in a two-phase decanter with a capacity of 500 kg/h (Oliomio, Toscana Enologica Morri, Italy). The produced oils were clarified by the natural sedimentation (two weeks) and the decantation to separate them from the remaining vegetable water and olive tissue particles. To determine the influence of the addition of olive leaves during industrial oil production on the composition and oxidation stability of the oil during storage, the Leccino cultivar fruits were processed into oil without (control oil) and with the addition of 2.5% of the olive leaves in three repetitions per treatment. For each repetition of processing, 300 kg of olive fruits were used. The produced oils were analyzed immediately after processing. From each of the three repetitions per treatment, two dark glass bottles with a volume of 0.25 L were filled and stored in the dark at controlled room temperature (18–22 °C) to examine the influence of the storage time (after 6 and 12 months) on the quality and composition of the produced oil samples.

### 2.2. Oil Analyses

#### 2.2.1. Quality Parameters

Oil quality parameters, free fatty acids (FFA) [40], peroxide value (PV) [41], and spectrophotometric indices (K_232_, K_270_, and ΔK) [42] were determined according to International Olive Council (IOC) standard methods.

#### 2.2.2. Fatty Acid Methyl Esters (FAME)

FAME analysis was performed according to IOC method [43] using a Varian 3350 gas chromatograph (Varian Inc., Harbour City, CA, USA) equipped with a flame-ionization detector and an Rtx-2.330 capillary column (Restek, Bellefonte, PA, USA).

#### 2.2.3. Pigments

Oil pigments, chlorophylls and carotenoids, expressed as pheophytin and lutein content (mg/kg oil) respectively, were determined colorimetrically according to the method of [44] using a UV/Vis spectrophotometer (Varian Cary 50, Varian, Harbor City, CA, USA).

#### 2.2.4. Oxidative Stability of the Oil

The oxidative stability of the oil was determined according to the AOCS official method [45] on the Oil Stability Instrument (Omnion, Decatur, IL, USA). The analysis was carried out in such a way that a stream of purified air passed through a sample of 5 g of oil, which was maintained at a constant temperature of 110 °C with an airflow of 120 mL/min. Waste air from the oil sample was then passed through a container containing deionized water, and the conductivity of the water was continuously monitored. The exhaust air contains volatile organic acids removed from the oxidizing oil, increasing water conductivity as the oxidation progresses. The oxidative stability index (OSI) is defined as the time, expressed in hours, required to achieve the maximum change in conductivity.

#### 2.2.5. Determination of Phenolic Compounds

Extraction and analyses of phenolic compounds in oil were according to the method of Jerman Klen et al. [46], modified by Lukić et al. [47] using an Agilent Infinity 1.260 HPLC system (Agilent Technologies, Santa Clara, CA, USA). Phenolic compounds were identified by comparing retention times and UV/Vis spectra with those of pure standards and those from Jerman Klen et al. [46]. Quantification using standard calibration curves or semiquantitative quantification of phenolic compounds was performed according to Lukić et al. [47].

#### 2.2.6. Total Phenolic Compounds

Total phenolic compounds in oils were extracted according to the protocol of Gutfinger [48] and determined according to the Folin–Ciocalteu colorimetric method using a UV/Vis spectrophotometer (Varian Cary 50, Varian, Harbor City, CA, USA). The results are expressed as mg of gallic acid equivalents per kg of oil.

#### 2.2.7. Volatile Compounds Determination

Volatile compounds in oil samples were isolated using headspace solid-phase microextraction (HS-SPME), following the modified method described in Brkić Bubola et al. [49] with modifications reported in Brkić Bubola et al. [50]. The volatile extraction was performed using SPME silica fiber divinylbenzene/carboxene/polydimethylsiloxane, length 1 cm, film thickness 50/30 μm (Supelco, Bellefonte, PA, USA). Varian 3350 gas chromatograph (Varian Inc., Harbor City, CA, USA), with a flame ionization detector and a capillary column Rtx-WAX (Restek, Bellefonte, PA, USA), was used for the analysis of volatile compounds. Identification was performed using a Varian 3900 GC coupled to a Varian Saturn 2100 T ion trap mass spectrometer (Varian Inc., Crawley, United Kingdom) equipped with the same column and using the same temperature program. Volatiles were identified by comparing compounds’ retention and mass spectra with those of pure standards and with mass spectra from the NIST05 library. Quantification was performed using calibration curves of pure standards: (*E*)-2-hexenal, 1-hexanol, (*Z*)-3-hexen-1-ol, (*E*)-2-hexen-1-ol, hexyl acetate, ethyl-2-methylbutyrate, (*E*)-3-hexen-1-ol, (*Z*)-2-hexen-1-ol, (*Z*)-2-penten-1-ol, (*E*)-2-penten-1-ol, (*E*)-2-pentenal, octanal, (*Z*)-3-hexenyl acetate, isovaleraldehyde, (*E*)-2-octenal, 3-pentanone, hexenal. Other volatiles were quantified semi-quantitatively using compounds with similar chemical structure for which standards were available.

#### 2.2.8. Sensory Analysis of VOOs

Sensory characteristics of oils were assessed by eight assessors trained for the sensory analysis of virgin olive oils according to the standard IOC method [51]. Differently from standard method, a modified evaluation sheet expanded with specific sensory characteristics (green grass/leaves, apple, tomato, almond, aromatic herbs, radicchio/arugula, green almond peel, sweet, and astringent) was used to more clearly show changes in oil sensory profiles. Furthermore, the complexity, harmony, and persistence of the oils were assessed using a 10-point overall structured rating scale from 0 (the lowest quality) to 10 (the highest quality).

#### 2.2.9. Determination of Oil Content, Extractability Index, and Oil Yield

Determination of oil content (%) in the olive paste samples was performed using Soxtec Avanti 2.055 apparatus (Foss Tecator, Höganäs, Sweden) [52].

Olive oil extractability index (EI) was determined according to Beltrán et al. [53] using the following Equation (1):

EI = (V × d)/(W × F) × 100,(1)

where V (mL) is the volume of extracted olive oil, d is the average density of olive oil (0.915 g mL^−1^), W (g) is the mass of olive paste, and F (%) is the oil content (on fresh weight).

Oil yield (%) was calculated [54] using the following, Equation (2):

Oil yield (%) = mass ratio extracted oil (g)/olive paste (g) × 100(2)

#### 2.2.10. Statistical Data Analysis

The chemical and sensory analysis results are presented as the mean value of the results of analyses determined in three repetitions ± standard deviation. The chemical and sensory analysis data were subjected to a one-way analysis of variance (ANOVA) at a significance level of 5%. Mean values were compared using Tukey’s honest significant difference test, *p* ≤ 0.05. Statistical data processing was performed using Statistica v. 13.2 (Stat-Soft. Inc., Tulsa, OK, USA). Additionally, data were processed using principal component analysis (PCA) using MetaboAnalyst v. 5.0 software [55]. Variables used for PCA were chemical (phenolic and volatile compounds) and sensory analysis data.

## 3. Results

### 3.1. Quality Parameters

The results of the basic quality parameters in Leccino cultivar oils produced in industrial conditions with and without the addition of olive leaves monitored immediately after processing (L-0) and through two periods of storage, after 6 months (L-6) and after 12 months (L-12) of storage, are shown in Table 1. The 2.5 addition of leaves had no significant effect on the content of FFAs compared to the control oils (Table 1), which is in agreement with the results of Di Giovacchino et al. [37] on the influence of leaf additions (1, 2, 3, and 5%) in Dritta and Leccino + Castiglionese cultivar oils, as well as with the results of Ammar et al. [29] on the influence of a 3% leaf addition in Chemlali and Chétoui cultivar oils. However, data on the increase of FFA in oils produced with the addition of olive leaves can be found in the literature [56]. In addition, Ammar et al. [29] found a trend of FFA increase in the oils of the Zalmati varieties and the Chemlali, and Zalmati cross-produced with 3% added leaf; a trend was also found by the authors of Malheiro et al. [9] for the influence of leaf addition (1, 2.5, 5, and 10%) in the oils of the Cobrançosa variety, but only in one year of two-year research. After 6 months of oil storage, no significant difference in FFA was found between oil produced with or without leaf additions. However, a slight increase in FFA value was found in oils stored for 12 months compared to the control oils (+10.53%) (Table 1), which indicates a slightly higher degree of hydrolytic deterioration of these oils. The reported increase in FFA content caused by the addition of leaves in oil processing is probably due to an increase in the presence of certain lipolytic enzymes that caused the hydrolysis of triacylglycerols, which increased the fatty acid release [9].

The results of PV, a quality parameter that indicates primary oil oxidation, have shown that the leaf addition had no significant effect on the primary oxidation in L-0-L and L-6-L oils compared to oils produced without the leaf addition (Table 1). In the literature published so far, contradictory data can be found on the influence of the leaf addition on PV. Some authors report that the leaf addition had no significant effect on PV [25,37,56], while others found an increase in PV values in oils produced with the leaf addition during production [9,29]. Malheiro et al. [9] explained the increase in PV in oils produced with the addition of leaves with the possible continuation of leaf respiration, which potentially increases the availability of oxygen during oil production and may favor the peroxidation process. However, after 12 months of storage, higher PV values (about 1 meq O_2_/kg) were found in oils produced with leaves compared to the control oils, which is not highly significant from the point of view of quality (Table 1) but indicates a slightly higher oxidation of oils produced with leaves.

Immediately after processing, the addition of olive leaves only slightly increased K_268_, a parameter that indicates the beginning of secondary oxidation in L-0-L oil (Table 1). Malheiro et al. [9] found a trend of increasing K-numbers under the influence of the addition of 1, 2.5, 5, and 10% of the leaves in Cobrançosa cultivar oil, but only in one of the two investigated years, while Sari and Ekinci [56] did not determine changes in K-numbers in Ayvalık cultivar oils produced with the addition of 2.5 and 5% of the leaves. After storage, in L-6-L and L-12-L oils, the addition of leaves had no significant effect on the increase in secondary oxidation products in the mentioned oils compared to control oils produced without the addition of leaves.

It can be concluded that the addition of leaves (2.5%) in the production of Leccino cultivar oil immediately after processing had a slight negative impact on oil quality parameters. Still, the observed oxidation and hydrolytic changes were higher after 12 months of storage. However, the quality of the produced oils was not significantly compromised since all determined values of quality parameters remained within the limits prescribed for the EVOO category [28].

### 3.2. Fatty Acids

In L-0-L oils produced on an industrial scale with olive leaves, only a mild diminish in the proportion of heptadecane (C17:0), linolenic (C18:3), and lignoceric acid (C24:0) were found compared to L-0-cont (Table 2). The decrease in C17:0 and C24:0 content is consistent with the results of Ammar et al. [29], who found the same trend in oils of the Chétoui cultivar produced with the addition of olive leaves (3%). The reduction of C24:0 was determined by Malheiro et al. [9] in only Cobrançosa cultivar oils produced with a 1% leaf addition as well as C18:3 reduction in the remaining three investigated cultivar oils (Chemlali, Zalmati, and the cross between Chemlali and Zalmati). Considering the previous study as well, it can be concluded that the composition of fatty acids in oils produced with the addition of leaves depends on the variety of olive fruits and leaves [29], the year of production, and the percentage of added leaves [9].

After storage, slightly higher proportions of stearic acid (C18:0) were found in L-6-L and L-12-L oils produced with the addition of leaves compared to the stored control oils. Since stearic acid is positively correlated with the oil oxidative stability [57], the recorded increase in stearic acid could probably contribute to the oxidative stability of L-6-L and L-12-L oils. In stored L-6-L oils, slightly lower proportions of linoleic (C18:2), linolenic (C18:3), eicosenoic (C20:1), and behenic acid (C22:0) were found compared to L-6-cont, while in L-12-L oils, these changes in the composition of fatty acids were not determined. Decreases in the proportion of C22:0 in the Chétoui cultivar oils produced with the addition of 3% of the leaves were also determined by Ammar et al. [29] and by Malheiro et al. [9] with the addition of 10% of the leaves in the Cobrançosa cultivar oils immediately after processing.

High oleic/linoleic acid (C18:1/C18:2) ratios were found in all oils in this study (Table 2), considering that values above seven indicate a high oxidative stability of the oil [20,58]. Immediately after processing and after 12 months of storage, no significant difference was found between the control oils and the oils produced with the addition of olive leaf regarding the oleic/linoleic acid ratio. Ammar et al. [29] also have not found a significant influence of leaf addition (3%) on this fatty acid ratio in four different monovarietal oils (Chemlali, Chétoui, Zalmati, and the cross between Chemlali and Zalmati).

It is important to emphasize that the proportions of fatty acids in the produced oils of the L cultivar with and without the leaf addition, before and after storage, were within the limits prescribed for EVOO [28]. Although there were slight changes in the proportions of individual fatty acids in the oils produced with the addition of the leaves, the total saturated, total monounsaturated, and total polyunsaturated fatty acids remained unchanged. These observations were consistent with the results of Ammar et al. [29], where the aforementioned groups of fatty acids remained unchanged after the addition of olive leaves (3%) in the production of four different varietal oils (Chemlali, Chétoui, Zalmati, and a crossbreed between Chemlali and Zalmati).

### 3.3. Pigments

**Figure 1:**
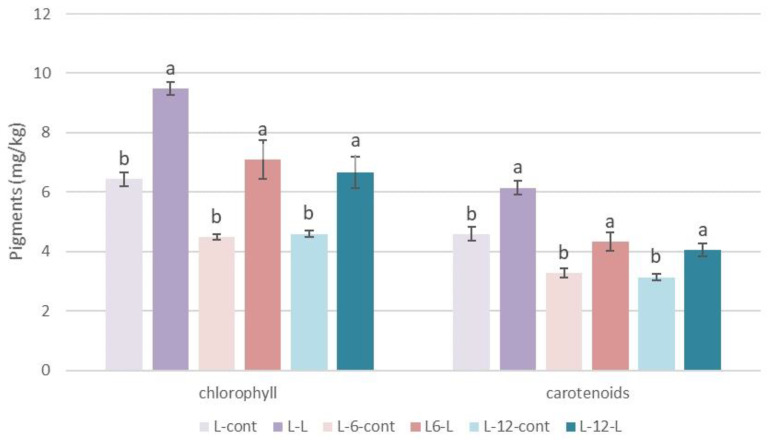
Pigments (mg/kg), chlorophyll and carotenoids, determined immediately after processing (L-0) and after 6 (L-6) and 12 (L-12) months of storage in oils of the Leccino cultivar (L) produced with (2.5%—L-L) or without (0%—L-cont) the addition of olive leaves. Results are expressed as mean values ± SD (*n* = 3). Means were compared separately for each storage time, and different small letters show significant difference (Tukey’s test. *p* ˂ 0.05) between oils produced with or without the addition of olive leaves.

A significant increase in chlorophyll (+47.59%) and carotenoid (+25.43%) concentrations in L-0-L oils compared to the control oils (Figure 1) was determined, probably due to the increased extraction of pigments from leaves whose leaf cells are destroyed during milling, and chlorophyll and pheophytin are released into the oil [37,59]. Several other authors [9,25,34,37] also found an increase in chlorophyll and carotenoids in oils after the addition of olive leaves. Higher pigment contents were maintained in oils produced with a leaf addition during storage (L-6-L and L-12-L) compared to oils produced without a leaf addition (Figure 1).

### 3.4. Oxidative Stability

**Figure 2:**
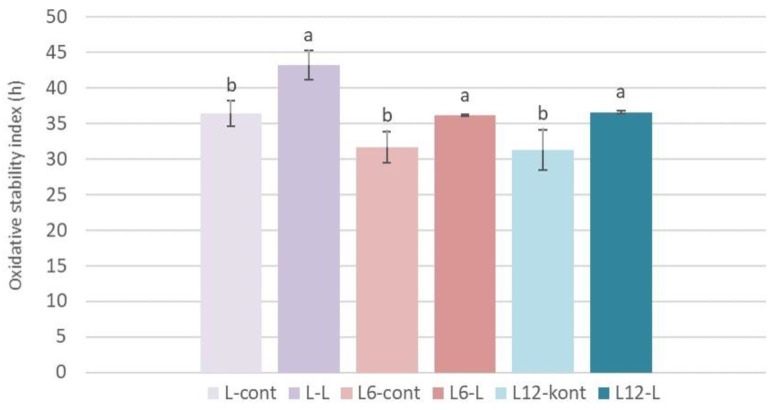
Oxidative stability index (hours) determined immediately after processing (L-0) and after 6 (L-6) and 12 (L-12) months of storage in oils of the Leccino cultivar (L) produced with (2.5%—L-L) or without (0%—L-cont) the addition of olive leaves. Results are expressed as mean values ± SD (*n* = 3). Meas were compared separately for each storage time, and different small letters show significant difference (Tukey’s test. *p* ˂ 0.05) between oils produced with or without the addition of olive leaves.

Figure 2 shows the oxidative stability index (OSI) determined in Leccino cultivar oils produced in industrial conditions with or without the addition of olive leaves during oil extraction, immediately after extraction, and after 6 and 12 months of storage (L-0, L-6 and L-12). The OSI increased in L-0-L oil by about +18.70% compared to the control oil produced without the addition of leaves. This result is in accordance with the results of other authors found in oils produced with the leaf addition in laboratory conditions [9,56] as well as under industrial conditions in Arbequina oils produced with a 1% leaf addition [26]. However, contradictory results of the influence of leaf additions on the oxidative stability of produced olive oils can be found in the literature. Di Giovacchino et al. [37] determined that the leaf addition did not significantly change the oxidative stability of the oil, while Ammar et al. [29] found that the oxidative stability of oil produced with the addition of olive leaves decreased compared to oil produced without a leaf addition.

After the 6 and 12 months of oil storage, a higher OSI value was found in the oils produced with the addition of leaves compared to the stored control oils (Figure 2). Oxidative stability is a very important parameter that provides a good estimation of the oil’s sensitivity to the oxidation process and its shelf life [9]. Therefore, the increase in the OSI indicates the positive influence of the leaf addition on the oil oxidative stability and the extension of the shelf life of the obtained product. The increment in the OSI is most likely connected to extracting antioxidant components from the leaves into oils [9]. Gutiérrez et al. [60] state that phenolic compounds are responsible for approximately 50% of the olive oil stability. The increase in oxidative stability results from the oxidative reaction of peroxidase and polyphenol oxidase with phenolic compounds in the presence of oxygen [56]. Therefore, the increase in the oxidative stability of oils produced with the addition of leaves can be associated with the increment of the total phenol content determined in these oils.

### 3.5. Total Phenols

**Figure 3:**
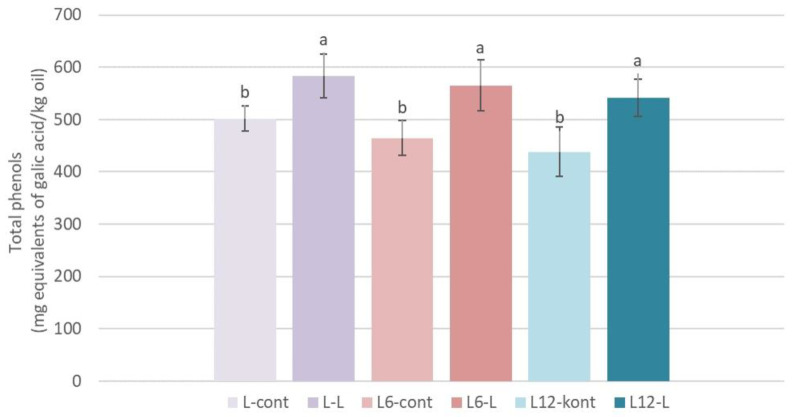
Total phenols (mg equivalents of galic acid/kg oil) determined immediately after processing (L-0) and after 6 (L-6) and 12 (L-12) months of storage in oils of the Leccino cultivar (L) produced with (2.5%—L-L) or without (0%—L-cont) the addition of olive leaves. Results are expressed as mean values ± SD (*n* = 3). Means were compared separately for each storage time, and different small letters show significant difference (Tukey’s test. *p* ˂ 0.05) between oils produced with or without the addition of olive leaves.

The addition of olive leaves in the amount of 2.5% in the industrial processing of Leccino cultivar oil increased (+29.55% compared to the control oil) the content of the total phenols immediately after processing (Figure 3), which is in accordance with the results of the authors Sevim et al. [33] in Memecik cultivar oil produced in laboratory conditions with the addition of 3% of the leaves. On the other hand, Sevim and Tuncay [34] did not determine a significant influence of adding olive leaves (1 and 3%) on the total phenols in the Ayvalık and Memecik cultivar oils. After storage, the higher content of total phenols was maintained in the oils produced with the addition of olive leaves (L-6 and L-12) compared to the stored control oils (Figure 3). Increased concentrations of the total phenols in oils produced with the addition of leaves most likely influenced the increase in the OSI (Figure 2) determined in the same oil samples.

### 3.6. Phenolic Compounds

Table 3 shows the results of concentrations of individual phenolic compounds determined in Leccino oils produced under industrial conditions with or without the addition of olive leaves immediately after processing and after storage. The addition of olive leaves influenced the increase in the concentration of total identified phenolic compounds in oils immediately after production (L-0-L, +29.55% compared to the control oils). Several previous studies also reported an increase in total phenolic compounds in oils of different cultivars produced with the addition of leaves [25,26,29,33], while some laboratory-scale research of oils produced with the leaf addition determined a decrease in the concentration of total identified phenolic compounds [32,35]. After storage, the concentration of total identified phenols remained higher in oils produced with a leaf addition (L-6-L and L-12-L).

In L-0-L oils, the concentrations of tyrosol, hydroxytyrosol, and hydroxytyrosol acetate and total simple phenols increased with the addition of leaves, which is in accordance with the results of Ammar et al. [38] in oils obtained with a 3% leaf addition for three of the four studied Tunisian olive cultivars. Marx et al. [35] concluded that Arbequina and Santulhana leaves’ incorporation in the malaxation process at the laboratory scale during Arbequina oil production may promote the formation of hydroxytyrosol and tyrosol formed due to an enzymatic or chemical hydrolysis pathway of oleuropein or ligstroside. After storage, the L-6-L and L-12-L oils retained higher concentrations of all simple phenols compared to the control oils.

The majority of individual secoiridoids and, consequently, the total secoiridoids concentration increased (+29.43% compared to control oils) in oils produced with the addition of olive leaves immediately after processing. In a study performed on the industrial scale, the concentration of total secoiridoids increased in the Arbequina cultivar oils produced with the addition of olive leaves [35]. In addition, Tarchoune et al. [25] found that the addition of 3% olive leaves affects the increase in the concentration of oleuropein derivatives in the oils of the Ouselati and Neb Jmel varieties, which is in accordance with the results of our research for 3,4-DHPEA-EDA (Table 3). The mentioned compound is present in olive leaves [61] and was most likely extracted from the leaf during the simultaneous grinding of the fruits and leaves as well as during malaxation of the olive paste together with the leaves. In contrast to these results, research conducted on a laboratory scale on the Buža oils [32] and Arbequina oils [35] with the leaf addition reported a decrease in the concentration of 3,4-DHPEA-EDA and total identified phenolic compounds compared to the control oils. In the present research, contrary to the recorded increase of most secoiridoids, oleuropein aglycone (isomer II) decreased (Table 3). After storage, the concentrations of most individual secoiridoids (3,4-DHPEA-EDA, oleuropein + ligstroside aglycones (isomers I and II), ligstroside aglycone (isomer II)) and total secoiridoids remained significantly higher in L-6-L and L-12-L oils produced with leaf additions compared to the stored control oils.

The addition of leaves during oil extraction influenced the increase in the concentration of total and individual flavonoids (luteolin, apigenin) in the oils immediately after processing (Table 3). These results are consistent with those of Tarchouna et al. [25], where an increase in total flavonoids was recorded in Neb Jmel and Oueslati oils with the addition of 3% of the olive leaves, while the authors Ammar et al. [38] found an increase in apigenin but a decrease in luteolin in oils with the addition of 3% of the leaves in all investigated oils obtained from four cultivars. Olive leaves contain flavonoids [62], which could be partially extracted into the olive paste and then into the produced oil during the fruit milling and malaxation of the olive paste. After storage, higher concentrations of luteolin, apigenin, and total flavonoids remained in L-6-L and L-12-L oils produced with the addition of leaves compared to the stored control oils.

Total and individual lignans (pinoresinol, acetoxypinoresinol) increased in L-0-L oils produced with the addition of leaves (Table 3), and higher concentrations were maintained in oils produced with the addition of leaves after storage. The increase in the concentration of the mentioned group of compounds can be linked to the fact that olive leaves contain lignans [63], which, due to their lipolytic character, more easily pass into the oil phase [64].

On the other hand, phenolic acids in the oils did not change significantly under the influence of adding olive leaves, and their concentrations remained stable even during storage. Consistent with our results, Tarchoune et al. [25] also found no significant influence of the 3% leaf addition during Ouselati and Neb Jmel cultivar oil extraction on the concentration of phenolic acids.

### 3.7. Volatile Compounds

The volatile compound concentrations determined in Leccino cultivar oils produced in an industrial condition with or without the addition of olive leaves during oil extraction are shown in Table 4. The addition of leaves slightly decreased the total identified volatile compounds (Table 4) immediately after processing (−8.48% compared to fresh control oils), while this difference was more pronounced after 12 months of storage (−25.37% compared to the stored control oils). Marx et al. [26] also observed a decrease in volatile compounds in Arbequina oil produced with the addition of 1% of the Arbequina olive leaves and explained the lack of volatile compound synthesis with the probably reduced activity of some enzymes participating in the lipoxygenase (LOX) pathway due to the addition of leaves to the olive paste during malaxation [35].

C6 volatile compounds, formed from linoleic and linolenic acid in the LOX pathway, are one of the most important volatile compounds responsible for the green odor characteristics of olive oils [65]. Total C6 volatiles (−10.48% compared to the control oils) and total aldehydes (−11.40%), as well as most individual aldehydes, decreased under the influence of leaf additions in L-0-L oil. Ammar et al. [29] also found a decrease in the total aldehyde concentration in the oil from the fruits of the crossbreed between Chemlali and Zalmati produced with the addition of 3% of the olive leaves. Marx et al. [35] reported a reduction of C6 volatile compounds in Arbequina oils produced on a laboratory scale with the addition of leaves of two cultivars. After 12 months of storage, the oils produced with the leaf addition remained at lower values of C6 volatiles and total aldehydes compared to the stored control oils.

The compound (E)-2-hexenal, responsible for the green fruity odor of olive oils [66] and considered a freshness marker in olive oils [67], was the most abundant compound in the volatile fraction of Leccino cultivar oils produced with and without the addition of olive leaves, although, with the addition of leaves, the concentration of the same compound was lower (a decrease of −11.78% compared to the control oil). Marx et al. [35] have also found the reduction of (E)-2-hexenal in the Arbequina oils produced at the laboratory scale with the addition of 1% of the leaves obtained from two cultivars. Contradictory results were reported during the extraction of Arbequina oils at an industrial scale with the addition of cv. Arbequina leaves (a decrease in (E)-2-hexenal compared to the control oil) and Santulhana cultivar leaves (an increase in (E)-2-hexenal compared to the control oil). Marx et al. [35] hypothesized that the leaf addition probably interferes with the enzymatic conversion of 13-L-hydroperoxides from linoleic acid, whose decomposition is catalyzed by hydroperoxide lyase, determining the formation of the (Z)-3-hexenal, in which the isomerization gives rise to (E)-2-hexenal. As a confirmation of this hypothesis, the concentrations of the compound (Z)-3-hexenal, responsible for the green odor of olive oils [66], in the present study were also lower in oils with the addition of olive leaves immediately after processing (−28.72% compared to the fresh control oil) and after 6 months of storage (−21.28% comparing to the stored control oil).

Concentrations of hexanal were slightly higher in oils produced with the addition of olive leaves immediately after processing (+15.90% compared to the fresh control oil). An increase in hexanal concentrations due to the influence of the addition of 3% of the olive leaves in the oils of the varieties Chemlali, Chétoui, and Zalmati were reported also by Ammar et al. [29]. After 6 months of storage, the hexanal concentration was still higher in oils with added leaves, while there was no difference in this compound between oils with and without added leaves after 12 months of storage. Hexanal is an aldehyde that can be formed in the LOX pathway from linoleic acid and is associated with the odors of green grass and apple [66] but also as a product of fatty acid oxidation [68,69,70]. Vichi et al. [70], however, consider that hexanal is not a reliable analytical marker of oxidation because it can be formed during the decomposition of hydroperoxides and through the LOX pathway and that oxidized oils cannot be distinguished from non-oxidized oils based on the concentration of hexanal only. Therefore, some authors suggested that a lower hexanal/E-2-hexenal ratio indicates better quality and a lower oil oxidation degree [71]. Following this criterion, it could be concluded that oils extracted with olive leaves have a higher degree of oxidation, especially after 12 months of storage (hexanal/E-2-hexenal ratio was 0.024 in L-12-cont vs. 0.035 in L-12-L), which is in agreement with previously discussed increased values of PV in L-12-L (Table 1).

However, immediately after processing, the concentrations of the total alcohols and most of the individual alcohols remained unchanged in the oils produced with the addition of leaves compared to the control oils, while the concentrations of individual alcohols decreased (1-hexanol, −22.63%, and (Z)-3-hexene-1-ol, −36.45%, compared to the control oil). This could be a consequence of the determined reduced amount of C6 aldehydes that are reducing into C6 alcohols via alcohol dehydrogenase in the LOX pathway, or it could be related to a possible decrease in the enzymatic activity of alcohol dehydrogenase in oils extracted with olive leaves. In contrast, Marx et al. [35] have found an increase in total C6 alcohols (especially (E)-2-hexen-1-ol) in Arbequina oils obtained with the addition of olive leaves at the laboratory level of oil production. After storage for 12 months, Leccino oils produced with the addition of leaves had lower concentrations of total C6 alcohols (−10.53%) compared to the control oils. The concentration of volatile compounds in virgin olive oils is closely related to the concentration and activity of enzymes in the olive fruit, and it also depends on the presence of enzyme activity inhibitors, such as phenolic substances [72]. The inhibitory effect of phenolic compounds on lipoxygenase activity from broccoli was confirmed by Baraniak and Krzepilko [73]. Since, in our research, the concentrations of phenolic compounds increased with the addition of leaves during processing (Table 3), it is possible that these compounds were one of the inhibitors of the LOX pathway enzyme activity involved in the formation of C6 volatile compounds.

**Figure 4:**
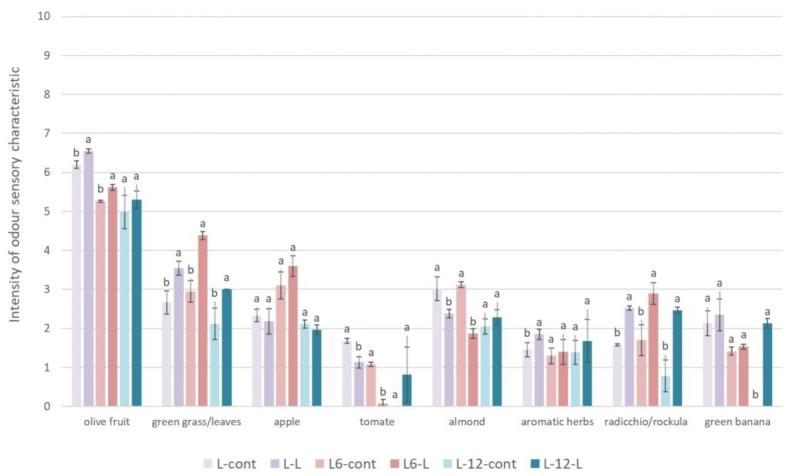
Results of quantitative descriptive sensory analysis of odor characteristics in Leccino oils produced with (2.5%—L-L) or without (0%—L-cont) the addition of olive leaves determined immediately after processing (L-0) and after 6 (L-6) and 12 (L-12) months of storage. Results are expressed as mean values ± SD (*n* = 3). Means were compared separately for each storage time, and different small letters show significant difference (Tukey’s test. *p* ˂ 0.05) between oils produced with or without the addition of olive leaves.

**Figure 5:**
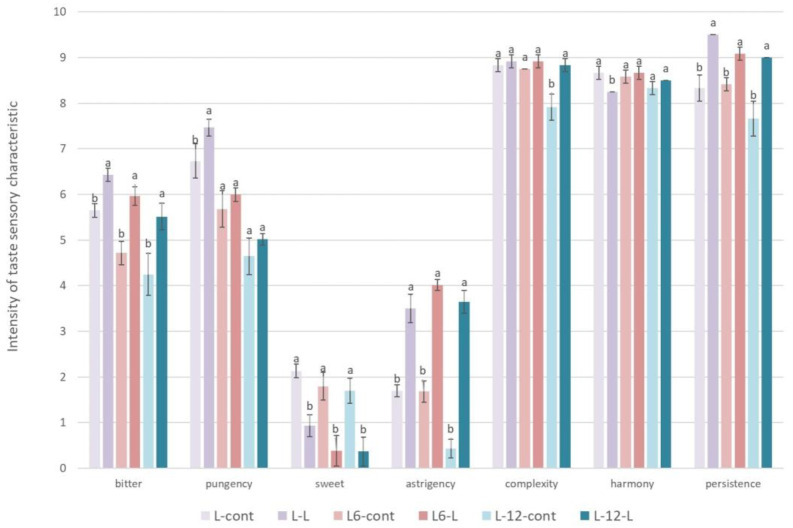
Results of quantitative descriptive sensory analysis of taste characteristics in Leccino oils produced with (2.5%—L-L) or without (0%—L-cont) the addition of olive leaves determined immediately after processing (L-0) and after 6 (L-6) and 12 (L-12) months of storage. Results are expressed as mean values ± SD (*n* = 3). Means were compared separately for each storage time, and different small letters show significant difference (Tukey’s test. *p* ˂ 0.05) between oils produced with or without the addition of olive leaves.

C5 volatile compounds, formed in an additional branch of the LOX pathway, also significantly contribute to the green aroma of olive oils [74]. The addition of olive leaves influenced the increase in the concentration of total C5 volatiles (+10.50% compared to the control oil) and individual ketones (3-pentanone, 1-penten-3-one) in L-0-L oil. Since 1-penten-3-one has a high odor activity value (OAV) in olive oils [49], higher concentrations of this compound can tentatively affect the increase in green odors of the oil as well as the intensity of the pungency and bitterness [66,74], while higher concentrations of 3-pentanone could be related to higher intensity of fruity odors with which this compound is associated [75], and all the sensory properties had higher intensities in olive oil with the leaf addition (Figure 4 and Figure 5). Marx et al. [35] have determined only one C5 volatile in Arbequina oils produced with the addition of olive leaves and have found no influence of leaf additions on the concentration of 2-penten-1-ol. On the other hand, after 12 months of storage, the concentration of total C5 volatiles and total ketones were lower (−10.38% and −8.83%, respectively, compared to the stored control oils) in oils with the addition of leaves.

### 3.8. Sensory Characteristics

The results of a sensory analysis on the olfactory characteristics in Leccino oils produced in industrial conditions with or without the addition of olive leaves during the oil extraction are shown in Figure 4. None of the investigated olive oils had a single negative characteristic. However, as far as the positive sensory characteristics are concerned, it is evident that olive leaf additions increased the intensity of the odor characteristics reminiscent of olive fruit, green grass/leaves, and radicchio/arugula in L-0-L oils, and increased intensities of the mentioned odor characteristics were retained even in the stored oils. The increase in these green odor characteristics could be associated with the increase in the concentration of certain volatile compounds responsible for the green odor [66,74], such as hexanal and C5 volatile compounds (Table 4). On the other hand, in the L-0-L and L-6-L oils, the addition of olive leaves reduced the tomato-like odor, which was probably influenced by the decrease in the concentration of (Z)-3-hexenal (Table 4), the volatile compound associated with tomato odor [76], and the decrease in almond odor, which can be explained by the decrease in the concentration of an almond odor-related volatile compound, (E)-2-hexenal [66], in the mentioned oils (Table 4).

Furthermore, Figure 5 shows the results of the taste characteristics of oils produced in industrial conditions with or without the addition of olive leaves during the oil extraction. In oils L-0-L, L-6-L, and L-12-L, it was determined that the leaf addition increased the bitter and pungent taste, astringency, and persistence of the oil. The increase in the intensity of taste characteristics in the oils produced with the addition of leaves was most likely influenced by the increased concentration of most individual and total secoiridoids and the total identified phenolic compounds in these oils (Table 3) [77,78]. Furthermore, in oils produced with the addition of olive leaves, a decrease in the sweet taste was found, and the reason for this could be an increase in the bitterness, pungency, and astringency in the mentioned oils. A sensory analysis revealed that the oils with the addition of leaves retained their complexity even after 12 months of storage, while the complexity decreased in the control oils. The harmony of the oil decreased slightly in oils after the leaf addition immediately after production, probably due to an increase in the astringency, bitterness, and pungency of the oil, which could disturb the overall impression of the oil harmony, but after storage, no significant difference between the treatments in harmony was found, probably due to a general decrease in taste characteristics in the oil during storage due to a decrease in phenolic compounds (Table 3).

**Figure 6:**
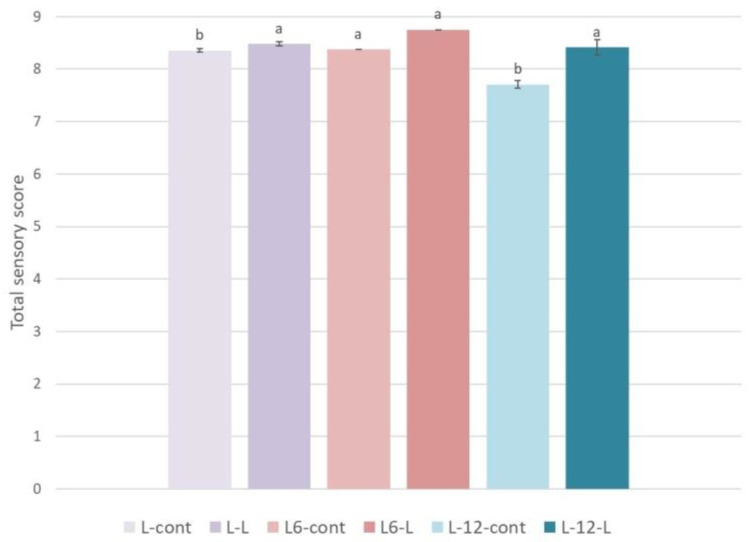
Total sensory score in Leccino oils produced with (2.5%—L-L) or without (0%—L-cont) the addition of olive leaves determined immediately after processing (L-0) and after 6 (L-6) and 12 (L-12) months of storage. Results are expressed as mean values ± SD (*n* = 3). Means were compared separately for each storage time, and different small letters show significant difference (Tukey’s test. *p* ˂ 0.05) between oils produced with or without the addition of olive leaves.

Immediately after production, a slight increase in the overall sensory rating of the produced oils with the addition of olive leaves (L-0-L) compared to the control oils was found, and a slightly higher overall rating was also found in oils L-6-L and L-12-L compared to the control oils (Figure 6). The aforementioned increase in the overall sensory rating is consequently the result of higher intensities of certain odors (olive fruit, green grass/leaves, radicchio/arugula) and taste (bitter, pungent, astringent, persistent) sensory characteristics determined in oils produced with the addition of olive leaves compared to the control oils.

### 3.9. Principal Component Analysis (PCA)

**Figure 7:**
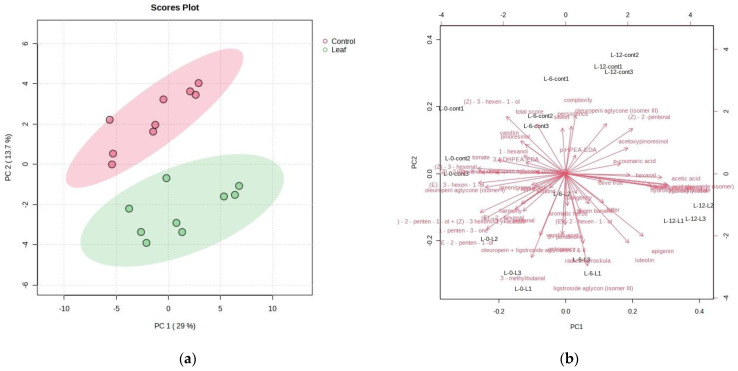
(**a**) Separation of Leccino cultivar olive oils according to the rate of leaf addition on PC1 and PC2 (red circles—oils produced without leaf addition (Control), green circles—oils produced with leaf addition (Leaf)). (**b**) Factor loadings of selected variables (the concentrations of individual phenolic compounds, the concentrations of individual volatile compounds, and the intensities of sensory attributes) along the directions of principal components PC1 and PC2.

The PCA allowed for a good differentiation of oil samples according to the two different factors: leaf addition and duration of storage. The dataset comprised 18 cases (oil samples) and 53 variables (concentrations of individual phenolic compounds, concentrations of individual volatile compounds, and the intensities of sensory attributes). The PCA allowed for a good differentiation of oil samples produced with (Leaf) and without (Control) a leaf addition (Figure 7a,b). The control oil samples were characterized mostly by tomato, sweet, persistence, complexity, pinoresinol, (Z)-3-hexenal, and 1-hexanol, which were loaded high on the negative sides of PC1 and on the positive side of PC2 in the second quadrant. Clearly, oil samples produced with a leaf addition (Leaf) were separated from the control samples by the intensity of green banana, pungency, bitter, astringency, harmony, and aromatic herbs as well as C5 volatile compounds responsible for green notes in the olive oils.

**Figure 8:**
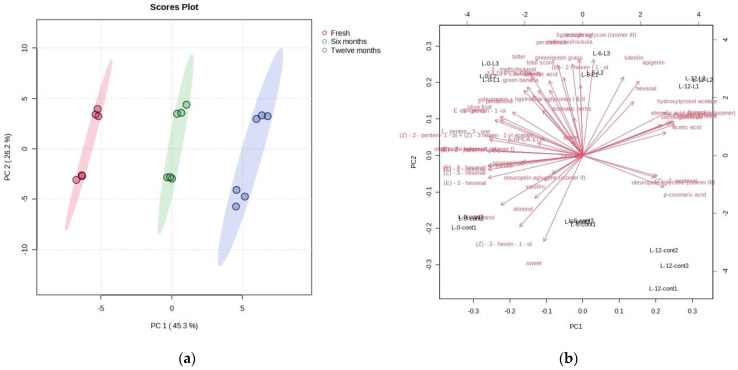
(**a**) Separation of Leccino cultivar olive oils according to the duration of oil storage on PC1 and PC2 (red circles—fresh oils produced with and without leaf addition (Fresh); green circles—oils produced with and without leaf addition, stored for 6 months (Six months); blue circles—oils produced with and without leaf addition, stored for 12 months (Twelve months)). (**b**) Factor loadings of selected variables (the concentrations of individual phenolic compounds, the concentrations of individual volatile compounds, and the intensities of sensory attributes) along the directions of principal components PC1 and PC2.

The PCA also allowed for a good differentiation of fresh and stored (6 and 12 months) oil samples regardless of the addition of leaves (Figure 8a,b). Fresh oil samples were clearly differentiated from the stored oil samples. Fresh oils were associated most strongly with the compounds in the negative PC1 values in the second and third quadrant, characterized mainly by most phenolic compounds, tomato, almond, green leaves/grass, green banana, bitter, and volatile compounds responsible for green notes. The samples stored for 6 months were characterized mainly by ligstroside aglicones, sweet, almond, and chicory/arugula. The samples stored for 12 months gravitated towards higher positive PC1 values and were characterized by lower amounts/intensities of the previously mentioned compounds and sensory characteristics as well as by increased values of variables with positive PC1 coordinates, such as hexanal, acetic acid, p-coumaric acid, and hydroxytyrosol acetate.

### 3.10. Oil Yield and Extractability Index

**Figure 9:**
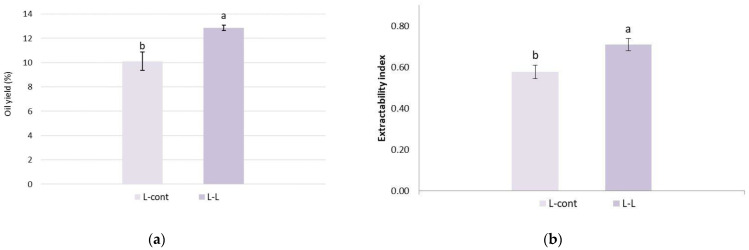
(**a**) Oil yield (%) and (**b**) extractability index of Leccino cultivar olive paste obtained with (2.5%—L-L) or without (0%—L-cont) the addition of olive leaves. Results are expressed as mean values ± SD (*n* = 3), and different small letters show significant difference (Tukey’s test. *p* ˂ 0.05) between treatments.

The oil yield and EI of Leccino cultivar olive paste obtained in industrial conditions with and without the addition of olive leaves during extraction is shown in Figure 9a,b. The obtained results indicate that the addition of olive leaves influenced the increase in the EI of the olive paste and, consequently, the oil yield (+27.17% compared to the control oil). Novoselić et al. [32] also found that 1–5% of leaf additions increases Buža cv. oil yields during laboratory oil extraction. On the other side, Sari and Ekinci [56] reported that the addition of olive leaves (2 and 5%) did not significantly affect oil yields in laboratory scale production, while the addition of 5% of the olive leaves even slightly reduced the EI due to the formation of an emulsion, which resulted in a decrease in the quantity of the extracted oil. The determined increase in EI and oil yields due to the addition of leaves in this study can be explained by the fact that the olive leaves played the role of a drainage material during the simultaneous milling of the fruits and olive leaves as well as during the malaxation of the olive paste in a similar way to the olive seeds creating canals in the olive paste, which facilitated the extraction of the oil [72].

## 4. Conclusions

The addition of olive leaves (2.5%) during the processing of Leccino cultivar olive fruits into oils in industrial conditions has a significant impact on the qualitative, nutritional, and sensory characteristics of freshly produced oils as well as oils stored for 6 and 12 months. Based on the obtained results, it can be concluded that the addition of leaves had only a slight negative impact on the quality parameters of the produced oil. Although there were slight changes in the proportions of individual fatty acids in the oils produced with the addition of the leaves, the proportions of total saturated, monounsaturated, and polyunsaturated fatty acids remained unchanged. The addition of leaves influenced the increase in the total phenolic concentration, secoiridoids, and pigments, and this trend was also maintained in oils after storage. The addition of olive leaves had a positive effect on the oxidative stability of oils. However, the addition of leaves had a decreasing effect on the C6 volatiles but an increasing effect on the C5 volatiles. The observed changes in volatile and phenolic compounds led to an increase in positive sensory characteristics in oils produced with the addition of leaves, especially in the taste characteristics, and the established improvements in sensory characteristics were maintained in the stored oils. From an economic point of view, adding olive leaves in industrial oil processing conditions positively affected the increase in the oil yield.

Therefore, it can be concluded that the addition of leaves (2.5%) during production in industrial conditions is a potential way to improve the quantitative, nutritional, and sensory characteristics of oils produced from olive fruits. The obtained results contribute to knowledge about the possibilities of enriching olive oils with phenolic and volatile compounds, and the results also contribute toward increasing the possible utilization of olive leaves and toward more sustainable olive production.

## Figures and Tables

**Table 1:** Quality parameters (free fatty acids—FFA, peroxide value—PV, spectrophotometric indices (K_232_, K_268_, and ΔK)) determined immediately after processing (L-0) and after 6 (L-6) and 12 (L-12) months of storage in oils of the Leccino cultivar (L) produced with (2.5%—L-L) or without (0%—L-cont) the addition of olive leaves.

Quality Parameters	L-0-cont	L-0-L	L-6-cont	L-6-L	L-12-cont	L-12-L	EVOO *
FFA (% of oleic acid)	0.14 ± 0.00	0.15 ± 0.00	0.14 ± 0.01	0.16 ± 0.01	0.17 ± 0.01 ^b^	0.19 ± 0.01 ^a^	≤0.80
PV (meq O_2_/kg)	4.20 ± 0.00	4.17 ± 0.06	6.30 ± 0.00	6.27 ± 0.06	7.00 ± 0.00 ^b^	8.00 ± 0.10 ^a^	≤20.0
K_232_	1.73 ± 0.10	1.86 ± 0.06	1.93 ± 0.03	2.06 ± 0.11	2.12 ± 0.03	2.21 ± 0.08	≤2.50
K_268_	0.13 ± 0.01 ^b^	0.15 ± 0.01 ^a^	0.14 ± 0.01	0.14 ± 0.01	0.15 ± 0.01	0.17 ± 0.01	≤0.22
ΔK	0.00 ± 0.00	0.00 ± 0.00	0.00 ± 0.00	0.00 ± 0.00	0.00 ± 0.00	0.00 ± 0.00	≤0.01

Results represent the mean ± SD (*n* = 3). The means marked with different letters in the same row of each individual storage period of Leccino oil are significantly different (Tukey’s test, *p* ˂ 0.05). * The extra virgin olive oil (EVOO) category limits [28].

**Table 2:** Composition of fatty acids (%) determined immediately after processing (L-0) and after 6 (L-6) and 12 (L-12) months of storage in oils of the *Leccino* cultivar (L) produced with (2.5%—L-L) or without (0%—L-cont) the addition of olive leaves.

Fatty Acid (%)	L-0-cont	L-0-L	L-6-cont	L-6-L	L-12-cont	L-12-L	EVOO *
Myristic (C 14:0)	0.01 ± 0.00	0.01 ± 0.00	0.01 ± 0.00	0.01 ± 0.00	0.01 ± 0.00	0.01 ± 0.00	≤0.03
Palmitic (C 16:0)	14.78 ± 0.19	14.83 ± 0.21	14.20 ± 0.12	14.38 ± 0.10	13.86 ± 0.12	13.89 ± 0.04	7.50–20.00
Palmitoleic (C 16:1)	1.49 ± 0.02	1.53 ± 0.04	1.40 ± 0.02	1.44 ± 0.03	1.28 ± 0.05	1.36 ± 0.05	0.30–3.50
Heptadecanoic (C 17:0)	0.04 ± 0.00 ^a^	0.03 ± 0.00 ^b^	0.04 ± 0.00	0.04 ± 0.00	0.04 ± 0.00	0.04 ± 0.00	≤0.40
Heptadecenoic (C 17:1)	0.09 ± 0.00	0.09 ± 0.01	0.09 ± 0.00	0.09 ± 0.00	0.10 ± 0.01	0.10 ± 0.00	≤0.60
Stearic (C 18:0)	1.82 ± 0.01	1.83 ± 0.01	1.81 ± 0.01 ^b^	1.83 ± 0.00 ^a^	1.81 ± 0.01 ^b^	1.83 ± 0.00 ^a^	0.50–5.00
Oleic (C 18:1)	74.27 ± 0.17	74.44 ± 0.22	75.10 ± 0.10	75.08 ± 0.09	75.78 ± 0.12	75.70 ± 0.04	55.00–85.00
Linoleic (C 18:2)	6.07 ± 0.19	5.86 ± 0.05	5.91 ± 0.02 ^a^	5.77 ± 0.04 ^b^	5.71 ± 0.04	5.69 ± 0.05	2.50–21.00
Linolenic (C18:3)	0.73 ± 0.01 ^a^	0.70 ± 0.01 ^b^	0.69 ± 0.01 ^a^	0.65 ± 0.00 ^b^	0.64 ± 0.01	0.62 ± 0.01	≤1.00
Arachidic (C 20:0)	0.29 ± 0.01	0.28 ± 0.01	0.31 ± 0.00	0.30 ± 0.01	0.32 ± 0.01	0.30 ± 0.01	≤0.60
Eicosenoic (C 20:1)	0.30 ± 0.01	0.29 ± 0.01	0.32 ± 0.00 ^a^	0.31 ± 0.01 ^b^	0.33 ± 0.01	0.32 ± 0.01	≤0.50
Behenic (C 22:0)	0.08 ± 0.00	0.08 ± 0.00	0.09 ± 0.00 ^a^	0.08 ± 0.00 ^b^	0.10 ± 0.00	0.09 ± 0.00	≤0.20
Erucic (C 22:1)	0.00 ± 0.00	0.00 ± 0.00	0.00 ± 0.00	0.00 ± 0.00	0.00 ± 0.00	0.00 ± 0.00	
Lignoceric (C 24:0)	0.04 ± 0.00 ^a^	0.03 ± 0.00 ^b^	0.04 ± 0.00	0.04 ± 0.00	0.05 ± 0.01	0.05 ± 0.00	≤0.20
∑SFA	17.05 ± 0.18	17.10 ± 0.21	17.05 ± 0.18	17.10 ± 0.21	16.18 ± 0.13	16.21 ± 0.03	
∑MUFA	76.15 ± 0.16	76.35 ± 0.19	76.15 ± 0.16	76.35 ± 0.19	77.49 ± 0.17	77.48 ± 0.05	
∑PUFA	6.80 ± 0.20	6.56 ± 0.04	6.80 ± 0.20	6.56 ± 0.04	6.34 ± 0.04	6.31 ± 0.05	
C18:1/C18:2 ratio	12.24 ± 0.39	12.71 ± 0.11	12.70 ± 0.04 ^b^	13.01 ± 0.07 ^a^	13.28 ± 0.11	13.30 ± 0.10	

The results represent the mean value ± SD (*n* = 3). Means marked with different letters in the same row for each individual storage period of Leccino oil are significantly different (Tukey’s test, *p* ˂ 0.05); SFA—saturated fatty acids, MUFA—monounsaturated fatty acids, PUFA—polyunsaturated fatty acids, * The extra virgin olive oil category (EVOO) limits [28].

**Table 3:** Phenolic compounds (mg/kg) determined immediately after processing (L-0) and after 6 (L-6) and 12 (L-12) months of storage in oils of the Leccino cultivar produced with (2.5%—L-L) or without (0%—L-cont) the addition of olive leaves.

Phenolic Compound (mg/kg) ^1^	L-0-cont	L-0-L	L-6-cont	L-6-L	L-12-cont	L-12-L
Simple phenols						
Tyrosol	3.37 ± 0.01 ^b^	5.00 ± 0.16 ^a^	5.28 ± 0.01 ^b^	7.28 ± 0.04 ^a^	7.32 ± 0.12 ^b^	8.86 ± 0.13 ^a^
Hydroxytyrosol	2.38 ± 0.13 ^b^	4.44 ± 0.12 ^a^	5.77 ± 0.18 ^b^	7.88 ± 0.20 ^a^	8.33 ± 0.14 ^b^	12.60 ± 0.63 ^a^
Hydroxytyrosol acetate ^S^	0.03 ± 0.01 ^b^	0.06 ± 0.01 ^a^	0.06 ± 0.01 ^b^	0.09 ± 0.01 ^a^	0.08 ± 0.01 ^b^	0.13 ± 0.02 ^a^
Vanillin	0.42 ± 0.01	0.41 ± 0.04	0.40 ± 0.02	0.38 ± 0.02	0.40 ± 0.02	0.39 ± 0.01
Total simple phenols	6.20 ± 0.13 ^b^	9.91 ± 0.26 ^a^	11.50 ± 0.17 ^b^	15.63 ± 0.18 ^a^	16.13 ± 0.07 ^b^	21.98 ± 0.78 ^a^
Secoiridoids						
Elenoic acid glucoside (isomer) ^S^	0.81 ± 0.01 ^b^	1.02 ± 0.03 ^a^	1.05 ± 0.07 ^b^	1.24 ± 0.05 ^a^	1.28 ± 0.07 ^b^	1.55 ± 0.13 ^a^
Oleacein (3,4-DHPEA-EDA) ^S^	303.34 ± 13.49 ^b^	436.78 ± 4.81 ^a^	258.35 ± 6.63 ^b^	315.13 ± 16.74 ^a^	237.97 ± 17.78 ^b^	287.23 ± 9.98 ^a^
Oleuropein aglycone (isomer I) ^S^	9.66 ± 0.15 ^a^	9.17 ± 0.36 ^a^	1.25 ± 0.06	1.14 ± 0.07	1.11 ± 0.07	1.12 ± 0.09
Oleochantal (*p*-HPEA-EDA) ^S^	176.28 ± 7.10 ^b^	190.58 ± 5.02 ^a^	163.60 ± 9.67	152.70 ± 7.58	165.34 ± 5.03	172.58 ± 2.22
Oleuropein + ligstroside aglycones (isomers I i II) ^S^	11.45 ± 0.31 ^b^	12.85 ± 0.46 ^a^	4.47 ± 0.23 ^b^	10.21 ± 0.21 ^a^	9.05 ± 0.20 ^b^	9.62 ± 0.22 ^a^
Oleuropein aglycone (isomer II) ^S^	9.91 ± 0.33 ^a^	8.43 ± 0.43 ^b^	8.73 ± 0.48	8.40 ± 0.63	8.33 ± 0.09 ^b^	9.15 ± 0.16 ^a^
Ligstroside aglycone (isomer II) ^S^	7.04 ± 0.43 ^b^	12.88 ± 0.18 ^a^	7.76 ± 0.19 ^b^	12.37 ± 0.25 ^a^	6.53 ± 0.29 ^b^	13.04 ± 0.45 ^a^
Oleuropein aglycone (isomer III) ^S^	2.50 ± 0.25	2.60 ± 0.46	2.60 ± 0.40	2.36 ± 0.13	3.84 ± 0.16	3.73 ± 0.27
Total secoiridoids	521.10 ± 15.88 ^b^	674.48 ± 8.75 ^a^	447.91 ± 9.24 ^b^	503.75 ± 17.93 ^a^	433.71 ± 21.29 ^b^	498.33 ± 12.73 ^a^
Flavonoids						
Luteolin	1.39 ± 0.06 ^b^	1.81 ± 0.16 ^a^	1.56 ± 0.06 ^b^	2.07 ± 0.10 ^a^	1.35 ± 0.32 ^b^	2.44 ± 0.22 ^a^
Apigenin	0.28 ± 0.03 ^b^	0.44 ± 0.00 ^a^	0.34 ± 0.02 ^b^	0.57 ± 0.01 ^a^	0.33 ± 0.05 ^b^	0.77 ± 0.03 ^a^
Total flavonoids	1.67 ± 0.09 ^b^	2.25 ± 0.16 ^a^	1.91 ± 0.07 ^b^	2.64 ± 0.12 ^a^	1.69 ± 0.34 ^b^	3.22 ± 0.25 ^a^
Lignans						
Pinoresinol	5.85 ± 0.30 ^b^	8.26 ± 0.29 ^a^	6.27 ± 0.57 ^b^	7.68 ± 0.29 ^a^	6.15 ± 0.24 ^b^	7.09 ± 0.03 ^a^
Acetoxypinoresinol ^S^	12.94 ± 0.30 ^b^	14.94 ± 0.14 ^a^	16.85 ± 0.67	17.58 ± 0.95	16.94 ± 0.29 ^b^	19.60 ± 0.32 ^a^
Total lignans	18.79 ± 0.41 ^b^	23.20 ± 0.42 ^a^	23.13 ± 1.15 ^b^	25.27 ± 0.67 ^a^	23.09 ± 0.24 ^b^	26.69 ± 0.31 ^a^
Phenolic acids						
Vanillic acid	0.27 ± 0.01	0.29 ± 0.02	0.29 ± 0.02	0.30 ± 0.01	0.30 ± 0.01	0.31 ± 0.02
*p*-coumaric acids	0.41 ± 0.01	0.40 ± 0.01	0.50 ± 0.02	0.45 ± 0.03	0.52 ± 0.03	0.50 ± 0.03
Total phenolic acids	0.68 ± 0.03	0.69 ± 0.02	0.79 ± 0.04	0.75 ± 0.03	0.84 ± 0.04	0.82 ± 0.05
Total phenolic content	548.43 ± 15.71 ^b^	710.53 ± 8.33 ^a^	485.23 ± 10.46 ^b^	548.03 ± 18.30 ^a^	475.17 ± 21.23 ^b^	544.63 ± 13.90 ^a^

^1^ The results represent the mean value ± SD (*n* = 3). Means marked with different letters in the same row of each individual storage period of Leccino oil are significantly different (Tukey’s test, *p* ˂ 0.05). ^S^ The phenolic compounds quantified semi-quantitatively.

**Table 4:** Volatile compounds (mg/kg) determined immediately after processing (L-0) and after 6 (L-6) and 12 (L-12) months of storage in oils of the Leccino cultivar produced with (2.5%—L-L) or without (0%—L-cont) the addition of olive leaves.

Volatile Compound (mg/kg) ^1^	L-0-cont	L-0-L	L-6-cont	L-6-L	L-12-cont	L-12-L
3-Pentanone	0.124 ± 0.006 ^b^	0.145 ± 0.006 ^a^	0.114 ± 0.009 ^b^	0.134 ± 0.001 ^a^	0.107 ± 0.006	0.101 ± 0.006
1-Penten-3-one	1.929 ± 0.016 ^b^	2.160 ± 0.058 ^a^	0.983 ± 0.084 ^b^	1.145 ± 0.055 ^a^	0.629 ± 0.010 ^a^	0.569 ± 0.025 ^b^
(*E*)-2-Penten-1-ol	0.860 ± 0.068	0.956 ± 0.040	0.689 ± 0.033 ^b^	0.853 ± 0.031 ^a^	0.640 ± 0.047	0.595 ± 0.025
(*Z*)-2-Pentenal	0.004 ± 0.003	0.000 ± 0.000	n.d.	n.d.	0.043 ± 0.013	0.037 ± 0.014
(*E*)-2-Pentenal	0.111 ± 0.008	0.109 ± 0.003	0.068 ± 0.003	0.070 ± 0.002	0.054 ± 0.005	0.047 ± 0.002
Hexanal	0.440 ± 0.027 ^b^	0.510 ± 0.021 ^a^	0.423 ± 0.045 ^b^	0.538 ± 0.041 ^a^	0.522 ± 0.017	0.546 ± 0.018
(*E*)-2-Hexenal	35.139 ± 1.105 ^a^	31.000 ± 1.063 ^b^	25.700 ± 1.163	23.373 ± 1.559	21.820 ± 0.690 ^a^	15.498 ± 0.961 ^b^
(*Z*)-2-Hexenal *	0.326 ± 0.013 ^a^	0.258 ± 0.010 ^b^	0.159 ± 0.002 ^a^	0.138 ± 0.007 ^b^	0.110 ± 0.003 ^a^	0.083 ± 0.003 ^b^
(*E*)-3-Hexenal *	0.134 ± 0.007 ^a^	0.109 ± 0.005 ^b^	0.058 ± 0.005 ^a^	0.047 ± 0.004 ^b^	0.037 ± 0.002 ^a^	0.023 ± 0.002 ^b^
(*Z*)-3-Hexenal *	0.094 ± 0.009 ^a^	0.067 ± 0.006 ^b^	0.047 ± 0.005 ^a^	0.037 ± 0.004 ^b^	n.d.	n.d.
1-Hexanol	0.380 ± 0.010 ^a^	0.294 ± 0.014 ^b^	0.313 ± 0.020 ^a^	0.255 ± 0.016 ^b^	0.264 ± 0.005 ^a^	0.173 ± 0.013 ^b^
(*E*)-3-Hexen-1-ol	0.014 ± 0.003	0.011 ± 0.001	0.010 ± 0.001	0.009 ± 0.001	n.d.	n.d.
(*Z*)-3-Hexen-1-ol	0.546 ± 0.049 ^a^	0.347 ± 0.015 ^b^	0.447 ± 0.054 ^a^	0.287 ± 0.024 ^b^	0.390 ± 0.039 ^a^	0.204 ± 0.014 ^b^
(*E*)-2-Hexen-1-ol	0.733 ± 0.037	1.186 ± 0.289	0.869 ± 0.027	1.175 ± 0.326	0.796 ± 0.043 ^b^	0.978 ± 0.077 ^a^
(*Z*)-2-Hexen-1-ol	n.d.	n.d.	n.d.	n.d.	n.d.	n.d.
3-Methylbutanal	0.057 ± 0.004 ^b^	0.090 ± 0.012 ^a^	0.038 ± 0.002 ^b^	0.066 ± 0.008 ^a^	0.038 ± 0.002 ^b^	0.051 ± 0.003 ^a^
(*Z*)-2-Penten-1-ol + (*Z*)-3-Hexenyl-acetat	1.859 ± 0.032 ^b^	2.012 ± 0.052 ^a^	1.329 ± 0.024 ^b^	1.500 ± 0.061 ^a^	1.181 ± 0.048 ^a^	1.000 ± 0.077 ^b^
Acetic acid *	0.007 ± 0.002	0.008 ± 0.005	0.023 ± 0.001	0.029 ± 0.006	0.049 ± 0.004 ^b^	0.118 ± 0.004 ^a^
Total ketones	2.053 ± 0.017 ^b^	2.305 ± 0.058 ^a^	1.097 ± 0.080 ^b^	1.279 ± 0.055 ^a^	0.736 ± 0.004 ^a^	0.671 ± 0.019 ^b^
Total aldehydes	35.690 ± 1.137 ^a^	31.620 ± 1.050 ^b^	26.191 ± 1.209	23.981 ± 1.521	22.396 ± 0.678 ^a^	16.090 ± 0.951 ^b^
Total alcoholes	3.532 ± 0.046	3.850 ± 0.293	2.968 ± 0.108	3.225 ± 0.331	2.631 ± 0.067 ^a^	2.354 ± 0.042 ^b^
Total C5 volatiles	4.772 ± 0.108 ^b^	5.273 ± 0.138 ^a^	3.115 ± 0.085 ^b^	3.632 ± 0.097 ^a^	2.557 ± 0.080 ^a^	2.266 ± 0.075 ^b^
Total C6 volatiles	37.252 ± 1.145 ^a^	33.349 ± 0.788 ^b^	27.762 ± 1.291	25.637 ± 1.237	23.792 ± 0.756 ^a^	17.399 ± 0.899 ^b^
Total volatiles	41.275 ± 1.195 ^a^	37.775 ± 0.799 ^b^	30.877 ± 1.256	29.269 ± 1.243	26.348 ± 0.685 ^a^	19.664 ± 0.948 ^b^

^1^ The results represent the mean value ± SD (*n* = 3). Means marked with different letters in the same row of each individual storage period of Leccino oil are significantly different (Tukey’s test, *p* ˂ 0.05); n.d.—not determined. * The volatile compounds quantified semi-quantitatively.

## Data Availability

Data are contained within the article.
